# The Book World of Medicine and Science

**Published:** 1904-08-06

**Authors:** 


### THE BOOK WORLD OF MEDICINE AND SCIENCE.

LECTURES ON CLINICAL PSYCHIATRY. By Dr. EMIL KRAEPELIN, of Munich. Revised and edited by THOMAS JOHNSTONE, M.D.Edin., M.R.C.P.Lond. (London: Baillière, Tindall and Cox. Demy 8vo. Pages 308. Price 10s. 6d. net.)

DR. KRAEPELIN recognises the impropriety of speaking of mental diseases because "in strictest terms we cannot speak of the mind as becoming diseased, whether we regard it as a separate entity, or as the sum total of our subjective experience;" but we feel sure that the solecism will be tolerated until something better can be found, and in the meantime we continue to use the old term. In the Introduction we are told that "all the insane are dangerous in some degree to their neighbours, and even more so to themselves. Mental derangement is the cause of at least a third of the total number of suicides." Perhaps most asylum medical men would be constrained to admit that the more they see of chronic so-called harmless lunatics the less they trust them; but in these days of unlocked doors and freedom it is questionable whether they will fully endorse Dr. Kraepelin's statement. The book consists of thirty lectures, each being devoted to the description of a different variety of insanity, and perhaps the most interesting chapters to the general practitioner are the first and the twelfth, the one dealing with melancholia and the other with the insanity which accompanies or follows acute disease. In the latter is drawn the distinction betwen the initial delirium of certain cases and the mental disturbances produced later by acute diseases, and it is very clearly pointed out that the former have a graver clinical significance than the latter. To prevent collapse in the initial delirium Dr. Kraepelin recommends the use of protracted baths, skilful feeding, and transfusions of common salt. Melancholia, we are reminded, is the "condition in which we see the gradual development of a state of apprehensive depression, associated with more or less fully-developed delusions. The most common of these are ideas of sin which generally have a religious colouring. Such are the ideas of having fallen away from God and being forsaken, or of being possessed by the devil. . . . Together with these are the apprehension of poverty, of having to starve, of being thrown into prison, of being brought before a Court, or even of execution." For treatment of this form of insanity, in addition to what we may term "general" treatment, "paraldehyde is generally to be recommended, or, under some circumstances alcohol, or occasional doses of trional. Opium is employed, to combat the apprehensiveness, in gradually increasing doses, which are then by degrees reduced. . . . Great care is needed in discharging patients." We have quoted these passages not only because they will show the reader the style of the lectures, but also as an example of the way the translation has been done. It would be impossible for anyone to study this work without having his powers of diagnosis considerably increased; but it is clear that one object, if not the chief object of diagnosis, is to lead up to treatment and prognosis. Hence in some of the sections we get less treatment than we should like, although, as Dr. Johnson says, it is never absent. The volume could hardly be recommended as the only one for a student to use when reading up for an examination; but it is certainly one which the teacher is almost bound to consult, and which the library of the alienist physician cannot afford to be without. Even the student would do well to read the graphic word pictures of insanity which it contains; and he will find phases of the disease described in a manner he is not likely to get elsewhere.

THE CARE AND FEEDING OF CHILDREN. By L. EMMETT HOLT, M.D., with an Introduction by ERIC PRITCHARD, M.D. Third Edition. (London: Sidney Appleton. 1904. Pp. 149. Price 2s. net.)

THIS little work is admirably adapted for those to whom it is addressed, viz., young mothers and children's nurses. The author's name is a sufficient guarantee of the soundness of his teaching. The method by which the teaching is conveyed, viz., a series of questions and answers, renders it precise and clear. The various problems raised and solved are just those which would occur to an intelligent mother or nurse, and we cannot help thinking that if a work of this nature were used as a text-book in every nursery, the health of the inmates would be better, and much unnecessary suffering would be avoided. The general care of an infant is discussed, attention being given to the bathing, the eyes, the mouth, the skin, the clothing, and the nursery. The instructions as to the feeding of an infant are simple, and easily carried out, and the author carefully avoids the elaborate manipulation of the milk, or the pseudo-scientific methods which have found favour across the Atlantic. In these days of experimental dietaries it is advantageous to have a safe guide as to the reliable foods for infants, and the author very wisely confines himself to these. The last part of the book consists of the description and treatment of many minor ailments, which cause considerable disturbance in the nursery circle, but which can easily be prevented or relieved.

A MANUAL OF AMBULANCE. By J. SCOTT RIDDELL, C.M., M.B, M.A. Fifth Edition. (London: Griffin and Co. 1904. Pp. 227, 21 full-page figures, and 156 Illustrations. Price 4s.)

MR. SCOTT RIDDELL, besides being a surgeon, is a teacher with wide and varied experience, and this experience he has brought to bear in compiling his manual. It is a book which will be found of great service to all those who study or teach first aid. The lecturer will find here useful hints as to the management of his class. For the student the emergencies usually treated in such a work have been fully and carefully dealt with, and, in addition, varying "electrical accidents" have been introduced and methods of rendering first aid advised. A special feature of the present edition of the book is the chapter devoted to "Ambulance Transport and Stretcher Drill," where the new stretcher drill is lucidly described and is further made clear by diagrams and plates. Those who know the weaknesses of a layman's therapeutics will notice with pleasure that here he is warned of the frequent danger of administering alcoholic stimulants, and has the aim and use of antiseptics explained to him. This is a useful book, and should serve its purpose well.

